# Does a carboxamide moiety alter the toxicokinetics of synthetic cannabinoids? A study after pulmonary and intravenous administration of *cumyl*-5F-P7AICA to pigs

**DOI:** 10.1007/s00204-024-03906-z

**Published:** 2024-12-04

**Authors:** Nadja Walle, Christiane Dings, Omar Zaher, Adrian A. Doerr, Benjamin Peters, Matthias W. Laschke, Thorsten Lehr, Michael D. Menger, Peter H. Schmidt, Markus R. Meyer, Nadine Schaefer

**Affiliations:** 1 Institute of Legal Medicine, Saarland University, Building 49.1, 66421 Homburg, Germany; 2 Department of Clinical Pharmacy, Saarland University, Building C5 3, 66123 Saarbrücken, Germany; 3 Institute for Clinical and Experimental Surgery, Saarland University, Building 65/66, 66421 Homburg, Germany; 4 Department of Experimental and Clinical Toxicology, Center for Molecular Signaling (PZMS), Saarland University, Building 46, 66421 Homburg, Germany

**Keywords:** Synthetic cannabinoids, *cumyl*-5F-P7AICA, Carboxamide, Pigs, Toxicokinetics, Allometric scaling

## Abstract

Synthetic cannabinoids (SCs) are consumed as an alternative to cannabis. Novel compounds are developed by minor modifications in their chemical structure, e.g. insertion of a carboxamide moiety as a linker, which can potentially lead to altered toxicokinetics (TK). Knowledge on the TK data of SCs, especially structural modified substances, is scarce. Hence, interpretation of toxicological results is challenging. Therefore, the aim of the present study was to evaluate the TK of *cumyl*-5F-P7AICA in a pig model, which was shown to be suitable for TK studies of SCs. A 200 µg/kg body weight dose of *cumyl*-5F-P7AICA was administered intravenously (n = 6) or inhalatively (n = 10) via an ultrasonic nebulizer to pigs. Blood specimens were repeatedly drawn over 6 h and the concentrations of *cumyl*-5F-P7AICA as well as its *N*-pentanoic acid (NPA) metabolite were determined using a fully validated LC–MS/MS method. Based on the concentration–time profiles, a population TK analysis yielded a three-compartment model for the TK of *cumyl*-5F-P7AICA, whilst a two-compartment model described the NPA best. The incorporation of transit compartments accounts for the time delay between the appearance of *cumyl*-5F-P7AICA and NPA in serum. Finally, the model was upscaled to humans using allometric scaling. In comparison to older SCs, a higher volume of distribution was determined for *cumyl*-5F-P7AICA. No further relevant differences of the TK properties were observed. Insertion of a carboxamide moiety into the chemical structure of SCs does not appear to have only minor influence on the TK.

**Supplementary Information:**

The online version contains supplementary material available at 10.1007/s00204-024-03906-z.

## Introduction

For several years, new psychoactive substances (NPS) have emerged on the drug market as synthetically produced variants of conventional drugs. These substances are included in herbal mixtures, bath salts or plant food (Elliott and Evans 2014) in forms of herbal smoking mixtures, powders or even liquids (Shafi et al. 2020). Due to the absence of clinical safety studies (Guirguis 2017), they are consumed without the understanding of potential consequences. Furthermore, consumers are unaware of the specific dose or the exact SCs involved, involuntarily becoming the ‘experimental subjects’. This can lead to unexpected and severe side effects, including e.g. nausea, vomiting, tachycardia, hallucinations and psychosis, which may result in life-threatening conditions (Hermanns-Clausen et al. 2013; Meyer 2016; Kraemer et al. 2019).

Even when isolated SCs and entire chemical structure elements are restricted, these regulations are circumvented by slight modifications in the chemical structures. Such modified SCs, e.g. those containing a carboxamide moiety, have gained increased attention since several years, as numerous intoxications and fatalities following their consumption have been reported (Oberhofer 2018; Kraemer et al. 2019; Giorgetti et al. 2020; Kleis et al. 2020; Zawadzki et al. 2021; Ferrari Júnior et al. 2022; de Oliveira et al. 2023; Walle et al. 2023; Houston et al. 2023).

**Fig. 1:**
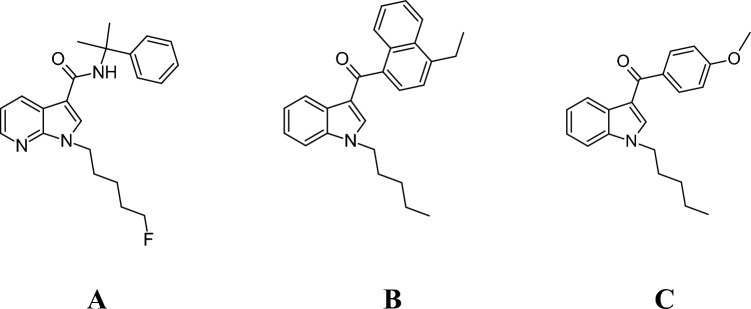
Molecular structures of *cumyl*-5F-P7AICA (**A**), JWH-210 (**B**), and RCS-4 (**C**)

*Cumyl*-5F-P7AICA (Fig. 1A) is one of those modified SCs and was first identified by the European Monitoring Centre for Drugs and Drug Addiction (EMCDDA) in 2015 (European Monitoring Centre for Drugs and Drug Addiction 2016). Besides a 7-azaindole core structure, *cumyl*-5F-P7AICA contains a carboxamide moiety as a linker between the core and the bridge residue and represents a further structurally modification of the SCs 5F‐CUMYL‐PICA and 5F‐CUMYL‐PINACA containing an indole or indazole core structure besides the carboxamide moiety (Banister et al. 2019). As a faster in vivo degradation via human carboxylesterases has been reported for various drugs (of abuse) containing amide moieties (Di 2018), structural modifications of SCs with incorporation of a carboxamide moiety could conceivably lead to altered toxicokinetic (TK) properties compared to ‘older’ SC containing e.g. a benzoyl moiety as a linker between the core and the bridge residue. However, in an in vitro metabolism study using various isoforms of recombinant human carboxylesterases with carboxamide containing SCs with different bridge residues, Wagmann et al. reported the degradation of SCs containing an ester moiety in the bridge residue, while the carboxamide linker remained stable (Wagmann et al. 2022).

In forensic toxicology, interpretation of plasma or serum concentrations is fundamental, e.g. for the evaluation of the driving ability of a person. For this purpose, human TK data of SCs are essential. However, due to ethical reasons, systematically controlled human studies are not possible. Therefore, TK data are predominantly available from in vitro studies, self-experiments or case reports, often lacking precise information on dose and time of intake or involving only a small number of participants (Castaneto et al. 2015; Meyer 2016; Houston et al. 2023). Furthermore, only limited TK data from systematically controlled animal studies have been published so far (Schaefer et al. 2015, 2016, 2017a, b, 2019; Castaneto et al. 2015; Walle et al. 2021, 2024; Doerr et al. 2021, 2024a, b). Thus, Schaefer et al. have successfully developed a pig model that can be used for the determination of the TK properties of (synthetic) cannabinoids and opioids after inhalative or intravenous (i.v.) administration (Schaefer et al. 2016, 2017a, b, 2018, 2019; Nordmeier et al. 2021). Compared to other in vivo setups, such as the zebrafish larvae or the rat model, the pig model offers various advantages: The larger blood volume enables a repeated sampling to study the TK. Additionally, pigs are considered to be very similar to humans with regard to the anatomical structures, isoenzymes, and physiological properties (Anzenbacher et al. 1998; Soucek et al. 2001; Meurens et al. 2012; Swindle et al. 2012).

In particular, only sparse in vivo TK data from systematically controlled studies on structurally modified SCs are available. Recently, Doerr et al. reported on the TK properties of the SC 5F-MDMB-P7AICA, which contains an ester moiety in the bridge residue in addition to a carboxamide linker (Doerr et al. 2024a).

Therefore, the aim of the present study was to elucidate the TK of *cumyl*-5F-P7AICA (Fig. 1A) after inhalative and i.v. administration using a sophisticated pig model. This drug was chosen as a representative for SCs containing a carboxamide moiety. In this study. the obtained data were used for the development of a TK model in order to predict human concentration–time profiles using allometric scaling. Subsequently, the results were compared with those previously published for the SCs JWH-210 (Fig. 1B) and RCS-4 (Fig. 1C) (Schaefer et al. 2016, 2018), which contain an indole core structure, to identify potential different TK properties due to the modified chemical structures.

## Materials and methods

### Chemicals, reagents, and preparations

A detailed list of the used chemicals and reagents as well as a description of the respective preparations (buffer solution, stock solutions, calibration standards, quality control samples as well as blank pig whole blood and serum) can be found in the Supplementary Information (SI).

### In vivo study

#### Animals

Analogous to previous studies (Walle et al. 2021, 2024), the in vivo experiments conducted in the present study were performed in accordance with the German legislation on protection of animals and the National Institutes of Health Guide for the Care and Use of Laboratory Animals (permission number: 44/2019).

Sixteen domestic male pigs of the Swabian Hall strain were used. The body weight (BW) of the pigs varied between 44 and 64 kg. In accordance to previous studies (Schaefer et al. 2018, 2019; Walle et al. 2021; Doerr et al. 2021; Nordmeier et al. 2022a, b), the animals had free access to tap water and daily standard chow up to 12 h before the start of the experiment. Then, they were kept fasting with still free access to water.

#### Surgical procedures

All surgical procedures were in accordance with already published previous studies (Schaefer et al. 2018, 2019, 2020; Walle et al. 2021; Doerr et al. 2021; Nordmeier et al. 2022a, b) and are described in detail in the SI.

#### Study design

The study included two different routes of administering the drug, i.v. or inhalative. First, six pigs received an i.v. dose of 200 µg/kg BW of *cumyl*-5F-P7AICA. For preparation of a solution with a concentration of 5 mg/mL, the SC was first diluted in ethanol. Following, to obtain the required dose of 200 µg/kg BW, the respective volume of the solution was withdrawn, fortified with 1 mL Polysorbat 80 for solubilization, and filled up with 0.9% sodium chloride to a final volume of 10 mL. Subsequently, the final solution was administered intravenously via the jugular vein over 30 s. Then, the venous catheter was washed for 30 s using 10 mL of 0.9% sodium chloride in order to remove possible retained substance (t = 0 min). After the washing step, blood samples were drawn 1, 2, 5, 10, 15, 30, 45, 60, 90, 120, 180, 240, 300, and 360 min after administration. Additionally, a control sample was taken before the administration of *cumyl*-5F-P7AICA.

Additional ten pigs received a 200 µg/kg BW dose of *cumyl*-5F-P7AICA via inhalative administration. For this purpose, *cumyl*-5F-P7AICA was initially dissolved in ethanol to obtain a stock solution of 5 mg/mL. The required volume to obtain a 200 µg/kg BW dose was diluted with ethanol to receive a final volume of 2 mL. The applied setup as well as the subsequent inhalative administration of *cumyl*-5F-P7AICA were in accordance to previous studies (Walle et al. 2021, 2024). Briefly, the prepared solution was nebulized and administered inhatively. For nebulization, the M-neb flow + ventilation ultrasonic nebulizer MN-300/7 (Nebutec, Elsenfeld, Germany) was used, applying the inspiration-triggered mode (< 0.2 mL/min). Blood samples were drawn prior to the administration as well as 1, 2, 5, 6, 7, 8, 9, 10, 15, 30, 45, 60, 90, 120, 180, 240, 300, and 360 min after the start of nebulization.

To obtain serum specimens, the blood specimens sampled during the experiment were centrifuged at 1476×*g* for 15 min. Blood and serum samples were stored at − 20 °C until analysis.

### Sample preparation

For qualitative and quantitative determination of *cumyl*-5F-P7AICA and its *N*-pentanoic acid (NPA) metabolite in pig blood and serum specimens, a solid phase extraction using Strata C18 endcapped cartridges (200 mg/3 mL; Phenomenex LTD, Aschaffenburg, Germany) was performed, following the procedure previously successfully applied for other SCs by Schaefer et al. (2015, 2016, 2018). If measured concentrations were above the calibration range, samples were diluted 1:10 and analyzed again. A detailed description of the sample preparation and method validation can be found in the SI.

### Liquid chromatography (LC)-quadrupole time of flight (TOF)–mass spectrometry (MS) apparatus

The settings of the LC-quadrupole TOF–MS used for detection and quantification of the substances in pig blood and serum samples were in accordance with a recent study (Walle et al. 2024) and can be found in detail in the SI.

### Non-compartmental analysis

A non-compartmental analysis (NCA) was performed using the Software R (Version 4.3.0, The R Foundation for Statistical Computing, Vienna, Austria) and R package ‘PKNCA’ (Version 0.10.2) (Denney et al. 2015). Mean values and standard deviations (SD) were calculated using the available parent drug and metabolite concentration measurements in serum and whole blood. The areas under the curve (AUCs) were derived from the concentration–time profiles using the linear up/log down method.

### Population TK modeling

A population (pop) TK model was developed using non-linear mixed-effects modelling techniques, facilitated by the software NONMEM (Version 7.4.3, ICON Development Solutions, Ellicott City, MD, USA). This approach enables the concurrent estimation of population medians for the model parameters alongside inter-individual (IIV) and residual variability. The model development process comprised three sequential phases: (I) Initial establishment of a TK model for *cumyl*-5F-P7AICA serum concentration after i.v. administration, involving exploration of various structural models (1-, 2-, 3- and 4 compartment models) and different elimination kinetics (i.e. linear and saturable processes); (II) subsequent integration of the metabolite formation into the parent model through an additional clearance rate from parent to metabolite, considering diverse structural models, metabolite formation and elimination kinetics; and (III) eventual extension of the model to incorporate parent and metabolite profiles following pulmonary administration by evaluating different absorption models. BW was incorporated as an exponential covariate on all clearance and volume of distribution parameters with an exponent of 0.75 to facilitate allometric scaling to human subjects (Schaefer et al. 2018).

For parameter estimation, the first-order conditional estimation algorithm with interaction was used. Model selection was based on visual inspection of goodness-of-fit plots (Karlsson and Savic 2007), precision of parameter estimates in the form of relative standard errors (Upton and Mould 2014), visual predictive checks (VPCs) and the objective function value (OFV) provided by NONMEM. Here, a nested model was considered superior if the difference of OFVs was > 3.84 points (chi^2^, p < 0.05, 1 df). For the VPC, 1000 simulations of the dataset were performed including random effects with the final model. Based on the simulation results, median serum concentration–time profiles and 90% prediction intervals were calculated and compared with the observed serum concentration. The software R (Version 4.3.0, The R Foundation for Statistical Computing) was used for the generation of the NONMEM dataset and graphics.

### Prediction of human exposure

The final pig model was upscaled to humans, using a reference BW of 70 kg and following allometric principles (Schaefer et al. 2018). Simulated scenarios included single-dose administrations of 0.5, 2 and 14 mg as well as multiple dose application of 2 mg every 60 min, for both i.v. and pulmonary application and with a fixed inhalation duration of 10 min. Each scenario underwent 1000 simulations including random effects. Subsequently, median simulated serum concentration–time profiles were plotted along with their corresponding 90% prediction intervals.

## Results

### Concentration–time profiles

**Fig. 2:**
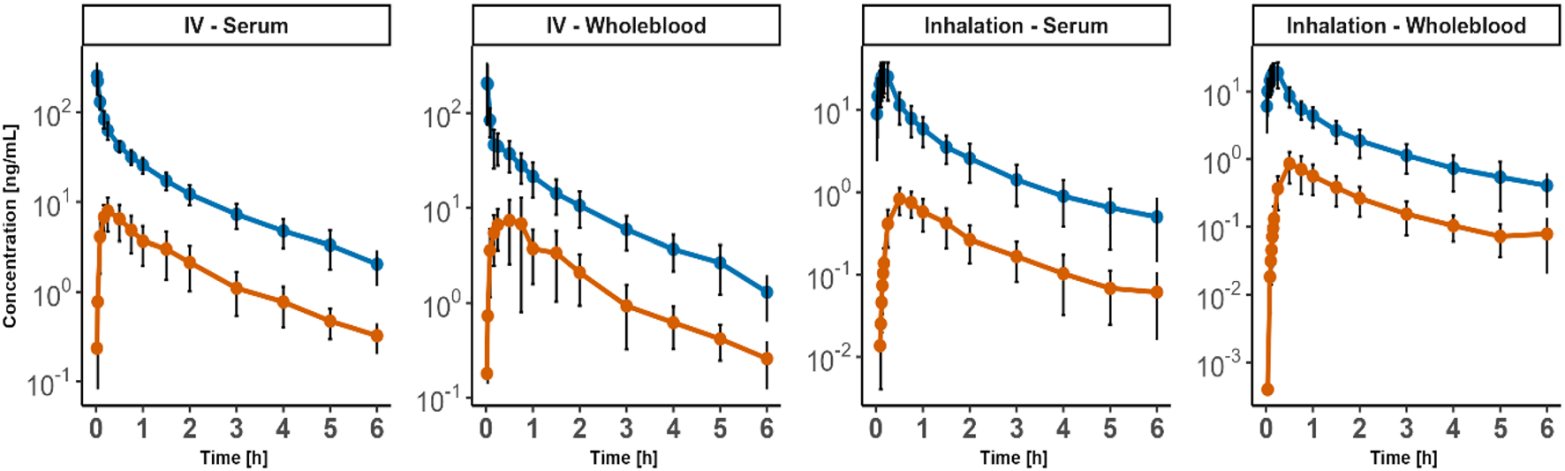
Mean serum and whole blood concentration–time profiles of *cumyl*-5F-P7AICA (blue lines) and its *N*-pentanoic acid metabolite (orange lines). Whiskers indicate standard deviations (color figure online)

The respective mean drug concentration of *cumyl*-5F-P7AICA and its NPA metabolite in pig serum and blood sampled during the experiment over 360 min after i.v. and inhalative administration are depicted in Fig. 2. Six pigs received *cumyl*-5F-P7AICA intravenously, yielding 83 measurements each for *cumyl*-5F-P7AICA and NPA. Ten pigs received *cumyl*-5F-P7AICA via pulmonary application, resulting in 180 and 156 measurements for *cumyl*-5F-P7AICA and NPA, respectively.

After i.v. administration of a 200 µg/kg BW dose of *cumyl*-5F-P7AICA, the maximum concentration (C_max_) of the parent substance in serum samples was found immediately after administration (t_max_ = 1 min) with 260 ± 99 ng/mL (mean ± SD; Fig. 2). Following, the concentrations declined rapidly to approximately 25 ± 5.1 ng/mL after 60 min. Afterwards, concentrations decreased slowly until the end of the experiment at 360 min. At this point of time, lowest concentrations (C_last_) were observed with 2.0 ± 0.87 ng/mL in serum specimens. Concerning the NPA metabolite, following a continuous increase, C_max_ was reached 15 to 30 min after administration with concentrations of 7.9 ± 3.2 ng/mL in serum. Afterwards, concentrations declined again to C_last_ of 0.32 ± 0.12 ng/mL in serum samples at 360 min.

On the other hand, after inhalative administration of the same dosage of *cumyl*-5F-P7AICA, C_max_ of 28 ± 11 ng/mL was reached after 10 min (t_max_) in serum samples (Fig. 2). Afterwards, concentrations fell rapidly to a mean concentration of 5.8 ± 2.3 ng/mL after 60 min. Subsequently, concentrations continued to decline, albeit to a lesser extent as after 360 min C_last_ was found to be 0.5 ± 0.36 ng/mL in serum. Regarding the NPA metabolite, maximum concentrations of 0.83 ± 0.30 ng/mL were reached in serum samples 30–50 min after drug administration (t_max_), declining to final concentrations at t = 360 min of 0.060 ± 0.050 ng/.

Figure 2 presents the mean concentration–time profiles after i.v. and pulmonary administration, which shows a three-phasic course for the parent, consisting of a tissue distribution (α) phase, with following elimination (β) phase, passing into a tissue release (γ) phase, and a two-phasic course for the metabolite.

The NCA revealed a half-life (t_1/2_) of 1.9 ± 0.52 h in serum for *cumyl*-5F-P7AICA after i.v. application. After inhalation, t_1/2_ amounted to 1.7 ± 0.36 h in serum. Bioavailability (F) in serum was 17 ± 13%.

For the NPA metabolite, t_1/2_ after i.v. administration was 1.4 ± 0.36 h and 1.7 ± 0.59 h after pulmonary administration for serum. All TK parameters obtained from the NCA are summarized in Table 1.

**Table 1:** Non-compartmental analysis summary

Parameter	Serum		Whole blood	
	I.V.	Inhalation	I.V.	Inhalation
	n = 6	n = 10	n = 6	n = 10
Body weight [kg]	51 ± 4.9	52 ± 6.3	51 ± 4.9	52 ± 6.3
***Cumyl*-5F-P7AICA**				
AUC_last_ [ng h/mL/kg]	99 ± 16	22 ± 10	75 ± 18	15 ± 5.8
AUC_inf_ [ng h/mL/kg]	104 ± 21	20 ± 9.0	80 ± 20	16 ± 6.2
C_max_ [ng/mL]	242 ± 90	26 ± 11	199 ± 137	20 ± 7.6
CL/F^a^ [L/min/kg]	0.030 ± 0.010	0.16 ± 0.060	0.040 ± 0.010	0.21 ± 0.070
V_z_/F^a^ [L/kg]	5.0 ± 1.2	23 ± 8.2	65 ± 4.2	33 ± 19
V_ss_/F^a^[L/kg]	3.2 ± 0.60	14 ± 5.2	3.9 ± 1.1	20 ± 8.6
t_1/2_ [h]	1.9 ± 0.52	1.7 ± 0.36	1.6 ± 0.70	2.0 ± 0.93
t_max_ [h]	0.020 ± 0.010	0.17 ± 0.040	0.030 ± 0.010	0.18 ± 0.040
F [%]	–	17 ± 13	–	21 ± 8.0
**NPA metabolite**				
AUC_last_ [ng h/mL/kg]	10 ± 4.7	1.3 ± 0.64	10 ± 5.5	1.3 ± 0.62
AUC_inf_ [ng h/mL/kg]	12 ± 4.5	1.5 ± 0.70	11 ± 5.5	1.5 ± 0.73
C_max_ [ng/mL]	7.9 ± 3.2	0.83 ± 0.30	8.8 ± 5.0	0.80 ± 0.44
CL/F^a^ [L/min/kg]	0.31 ± 0.16	2.3 ± 1.5	0.31 ± 0.20	2.5 ± 1.5
V_z_/F^a^ [L/kg]	37 ± 31	310 ± 249	36 ± 35	351 ± 235
V_ss_/F^a^ [L/kg]	34 ± 21	329 ± 246	33 ± 25	370 ± 306
t_1/2_ [h]	1.4 ± 0.36	1.7 ± 0.59	1.4 ± 0.38	1.8 ± 0.34
t_max_ [h]	0.25 ± 0.00	0.84 ± 0.30	0.56 ± 0.52	0.53 ± 0.11

*AUC*_*last*_ area under the curve calculated from zero to last observation, *AUC*_*inf*_ area under the curve from zero to infinity, *CL* clearance, *C*_*max*_ maximum observed concentration, *F* bioavailability, *n* number of animals, *t*_*1/2*_ elimination half-life; results are presented as mean (± standard deviation)

^a^Apparent clearance and apparent volume of distributions (CL/F, V_z_/F, V_ss_/F) for parameters derived after inhalation

### PopTK model

A popTK model was developed to examine serum concentration–time profiles of *cumyl*-5F-P7AICA and its NPA metabolite following pulmonary or i.v. application. The modeling analysis revealed that a three-compartment model with linear clearance provided the best description of the serum concentration–time profiles of *cumyl*-5F-P7AICA, while a two-compartment model was optimal for describing the profiles of the NPA metabolite. For pulmonary application, the dose was administered as a bolus to an absorption compartment, which was emptied after the individually recorded inhalation duration. The bioavailability was estimated to be 48%. However, considering the emptying of the inhalation compartment, the actual bioavailable fraction is lower.

Due to insufficient knowledge regarding the fraction of the parent metabolized to NPA, it was not possible to simultaneously determine the fraction of the parent metabolized to NPA and the volume of distribution of NPA. Consequently, the central volume of distribution of NPA (VM) was fixed to that of *cumyl*-5F-P7AICA (V). The clearance rate to the metabolite NPA was expressed as a fraction of the total clearance rate. With these assumptions, the fraction metabolized to NPA after i.v. and inhalative administration revealed 24 vs. 12%, respectively. Transit compartments were incorporated to account for the delay between the appearance of *cumyl*-5F-P7AICA and NPA in serum. Here, one transit compartment sufficed to describe the data after i.v. administration whereas two compartments were required after pulmonary application, resulting in mean transit times of 6.4 and 12 min after i.v. and pulmonary application, respectively. A schematical representation of the model can be found in Fig. SI 1.

Model parameters were estimated with sufficient precision (RSE < 45.9%). IIV was observed in various parameters, comprising the bioavailable fraction after inhalation (F), *cumyl*-5F-P7AICA clearance (CL), the fraction metabolized to NPA (f_MET_), the transit rate (ktr), the central volume of distribution (V) and intercompartmental clearances of *cumyl*-5F-P7AICA and NPA (Q1, Q2, QM). All parameter estimates can be found in Table 2. The associated differential equations can be found in the SI.

**Table 2:** Model parameter estimates

Parameter	Description	Unit	Estimate	RSE
***cumyl-*5F-P7AICA**				
Ka	First-order absorption rate from lung compartment	min^−1^	0.051	19%
F	Bioavailable fraction after inhalation	–	0.48	16%
CL	Clearance from central compartment	L/min/kg^0.75^	0.092	6.9%
V	Volume of the central compartment	L/kg^0.75^	1.5	16%
V_P1_	Volume of the peripheral compartment	L/kg^0.75^	4.4	5.8%
Q1	Intercompartmental clearance	L/min/kg^0.75^	0.030	16%
V_P2_	Volume of the peripheral compartment	L/kg^0.75^	3.3	4.5%
Q2	Intercompartmental clearance	L/min/kg^0.75^	0.21	18%
V_ss_	Volume of distribution at steady-state	L/kg^0.75^	9.2	
t_1/2α_	Elimination half-life during the alpha phase	min	2.6	–
t_1/2β_	Elimination half-life during the beta phase	min	32	–
t_1/2γ_	Elimination half-life during the gamma phase	min	146	–
IIV F	Interindividual variability F	%CV	64	36%
IIV CL	Interindividual variability CL	%CV	24	18%
IIV V	Interindividual variability V	%CV	60	17%
IIV Q1	Interindividual variability Q1	%CV	49	12%
IIV V2	Interindividual variability V2	%CV	69	19%
PRE	Proportional residual error	%	9.0	9.2%
ARE	Additive residual error (fixed)	ng/mL	0.010	–
***N*-pentanoic acid metabolite**				
f_MET_	Fraction metabolized from cumyl-5F-P7AICA to NPA	–	0.24	24%
f_APP_	Fractional change of f_MET_ for pulmonary application	–	− 0.48	22%
ktr	Transit rate between cumyl-5F-P7AICA and NPA	min^−1^	0.15	20%
CL_M_	Clearance from central compartment	L/min/kg^0.75^	0.18	19%
V_PM_	Volume of the peripheral compartment	L/kg^0.75^	8.2	27%
QM	Intercompartmental clearance	L/min/kg^0.75^	0.024	45%
IIV f_MET_	Interindividual variability f_MET_	%CV	43	12%
IIV ktr	Interindividual variability ktr	%CV	66	18%
IIV QM	Interindividual variability QM	%CV	126	25%
PRE	Proportional residual error	%	18	12%
ARE	Additive residual error (fixed)	ng/mL	0.010	–

**Fig. 3:**
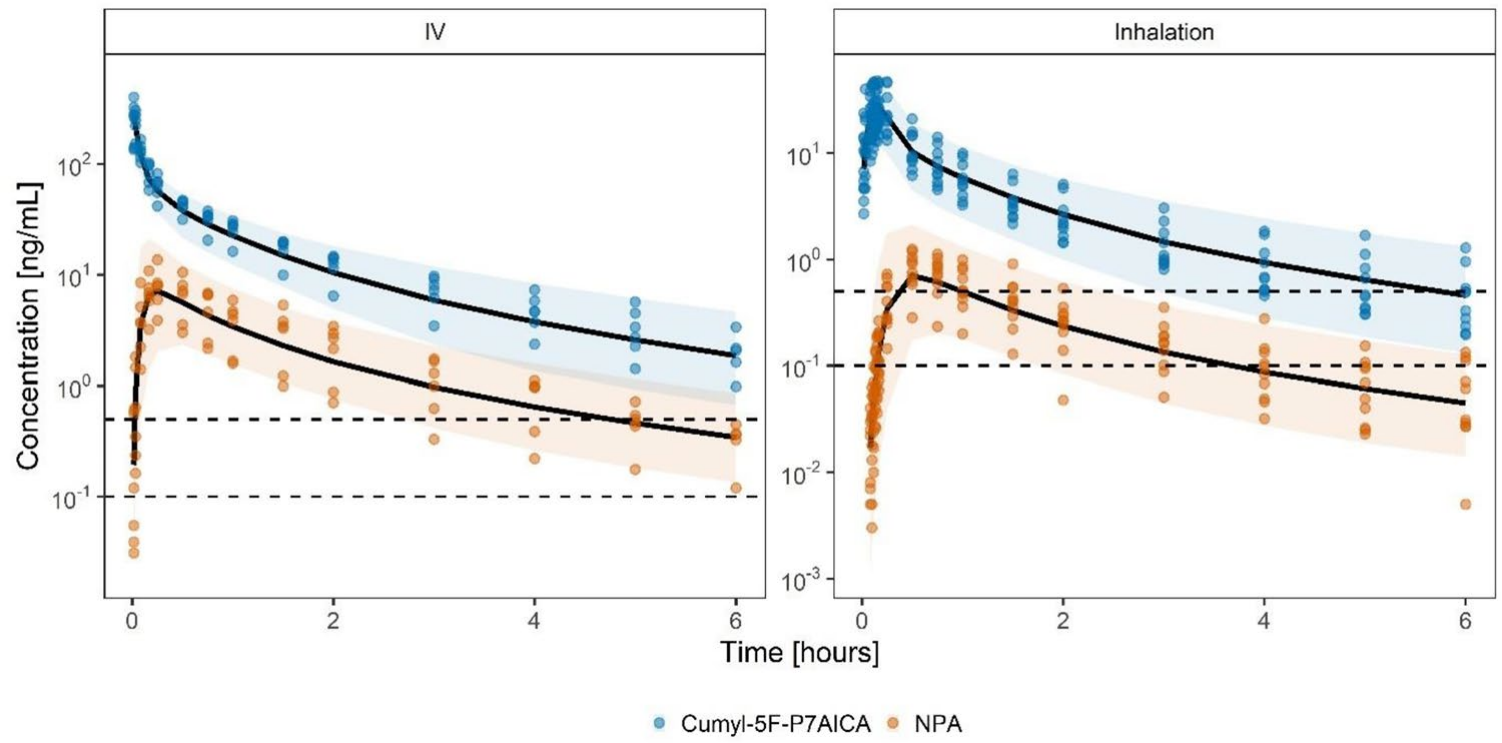
Visual predictive checks (VPCs) stratified by analyte and application. Observed serum concentrations are represented as circles. Lines represent the simulation median. Bands represent the simulated 90% prediction intervals. Dashed lines represent the LLOQ

Goodness-of-fit plots (Fig. SI 2) demonstrate a good agreement between observed data and model predictions, while VPCs (Fig. 3) confirm the model’s descriptive performance for both application forms without bias and with appropriate variability. Observed and predicted individual serum concentration–time profiles further illustrate the accurate depiction of observed concentrations (Fig. SI 3).

### Prediction of human exposure

**Fig. 4:**
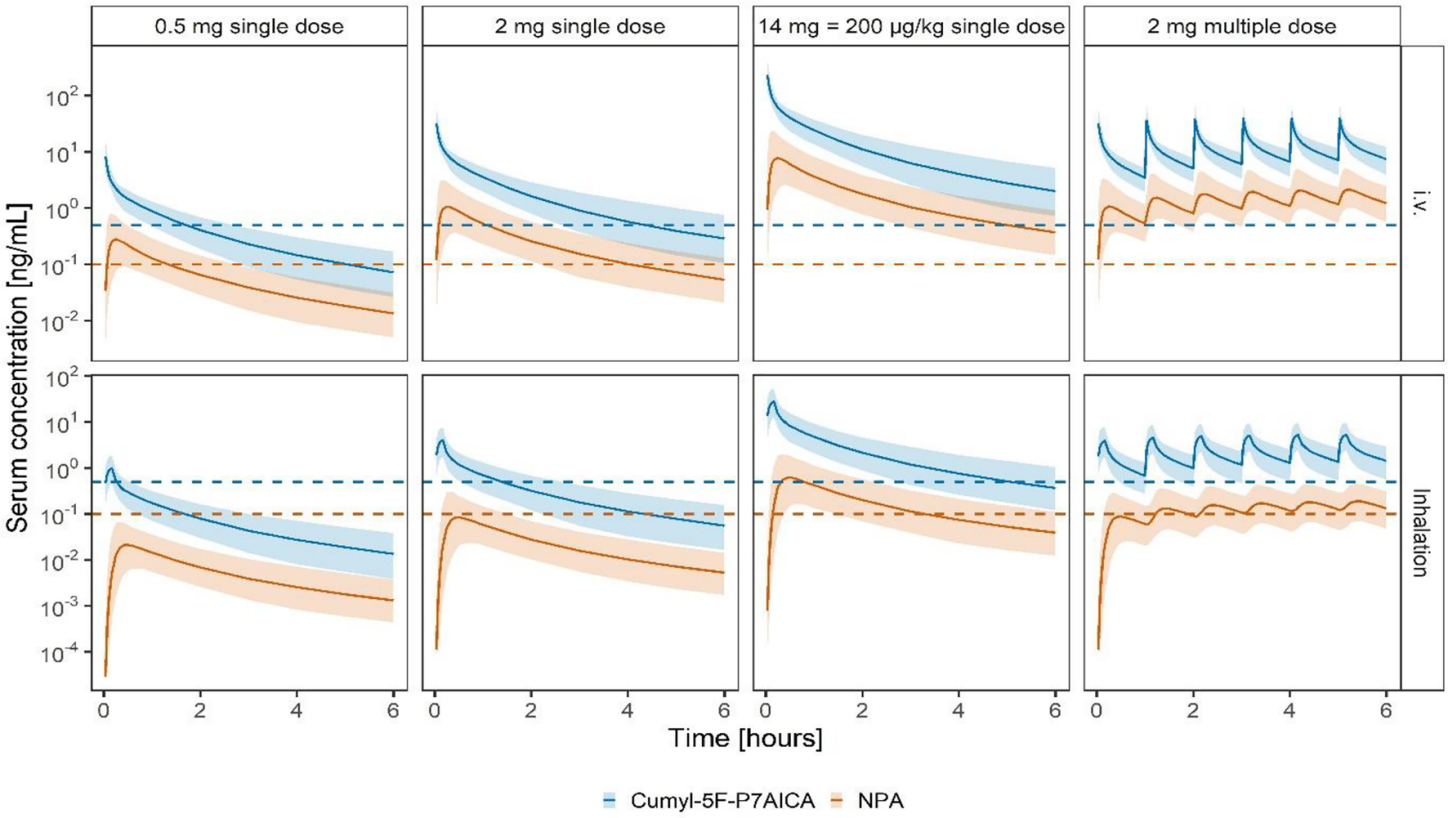
Prediction of human serum concentration–time profiles after i.v. application and inhalation of various doses. Full lines represent the simulation median. Bands represent the simulated 90% prediction intervals. Dashed lines represent the LLOQ

The human exposure to *cumyl*-5F-P7AICA and its NPA metabolite was predicted based on the popTK model developed for pigs using allometric scaling for BW. Figure 4 depicts serum concentration–time profiles for a 70 kg human after the i.v. application and inhalation of 0.5, 2 and 14 mg *cumyl*-5F-P7AICA as a single dose and 2 mg as a multiple dose every 60 min. Inhalation duration was set to 10 min.

## Discussion

### Dosage

In the present TK study, a dose of 200 µg/kg BW *cumyl*-5F-P7AICA was given i.v. or inhalatively to the animals. Depending on the respective BW of the pigs, the administered dose resulted in a total administered quantity of 8.8–12.8 mg *cumyl*-5F-P7AICA. This dosage was chosen based on previous TK studies demonstrating that a 200 µg/kg BW dose of *cumyl*-5F-P7AICA as well as other various SCs is well tolerated by pigs (Schaefer et al. 2016, 2018; Walle et al. 2021; Doerr et al. 2021). Additionally, 200 µg/kg BW is within the range of SC doses suggested by drug users in drug fora (Eve & Rave 2012). Thus, the dose administered in the present work reflects an authentic setting.

### Concentration–time profiles of *cumyl*-5F-P7AICA and its NPA metabolite

After i.v. administration, substantially higher concentrations of *cumyl*-5F-P7AICA as well as its NPA metabolite were detected compared to inhalative administration. As anticipated, C_max_ of the parent compound was observed immediately following i.v. administration, whereas with inhalative administration C_max_ was reached after a 10 min delay. The bioavailable fraction after inhalative administration obtained from the NCA amounted to 17 and 21% based on serum and whole blood data, respectively. A number of reasons are responsible for this low estimation: For one, an in vitro drug delivery efficiency test revealed that only 74 ± 10% of the nebulized SC dose reaches the lungs. Furthermore, several ‘lung defense mechanisms’, such as mechanical or chemical barriers (e.g. surfactant, proteolytic enzymes), as well as a pulmonary first-pass effect and metabolism of the inhaled substances can occur (Bend et al. 1985; Bakhle 1990; Boer 2003; Newman 2017).

The obtained serum concentration–time profiles as well as the TK properties of *cumyl*-5F-P7AICA are similar to other SCs after i.v. or inhalative administration of JWH-210 and RCS-4 to pigs (Schaefer et al. 2016, 2018). C_max_ (260 ± 99 ng/mL) and C_last_ (0.32 ± 0.12 ng/mL) obtained in the present work for *cumyl*-5F-P7AICA were slightly lower compared with RCS-4 (316 ± 60 ng/mL (C_max_) and 3.8 ± 1.1 ng/mL (C_last_)) after i.v. administration (Schaefer et al. 2016). For JWH-210, substantial higher C_max_ values (1600 ± 362 ng/mL) were determined after i.v. administration (Schaefer et al. 2016). After inhalative administration of 200 µg/kg BW of JWH-210 and RCS-4 to pigs, mean C_max_ values of 34–66 ng/mL 10–15 (t_max_) after start of nebulization were reported (Schaefer et al. 2018). Furthermore, 8 h after substance administration, mean concentrations of 0.87–1.5 ng/mL of the investigated parent substances were determined. Regarding the metabolites, Schaefer et al. reported on a C_max_ range between < 1.0–11 ng/mL with t_max_ between 5–60 min depending on the respective metabolite after inhalative administration (Schaefer et al. 2018). The data obtained in the current study for *cumyl*-5F-P7AICA and the NPA metabolite is in a good agreement with these values, with 28 ± 11 ng/mL and 0.83 ± 0.30 ng/mL (C_max_) 10 min and 30–50 min (t_max_) after drug administration as well as C_last_ values of 0.5 ± 0.36 ng/mL and 0.060 ± 0.050 ng/mL, respectively. Nevertheless, C_last_ of *cumyl*-5F-P7AICA in serum is considerably lower than those of JWH-210 and RCS-4 suggesting a faster in vivo elimination. In comparison, the C_max_ and t_max_ values of the NPA metabolite are in the same range as those reported by Schaefer et al. (Schaefer et al. 2018) for the metabolites of JWH-210 and RCS-4.

In addition, Doerr et al. recently reported on the TK properties of the SC 5F-MDMB-P7AICA after inhalative administration using the pig model (Doerr et al. 2024a). This substance also represents a structurally modified SC, containing a carboxamide moiety in the linker as well as an ester moiety in the bridge residue (Doerr et al. 2024a). Additionally, an NCA as well as a popTK analysis were performed for 5F-MDMB-P7AICA analogously to the present study. A similar serum concentration–time course was found for 5F-MDMB-P7AICA compared to the data obtained for *cumyl*-5F-P7AICA, with C_max_ values of 63 ± 18 ng/mL 6–10 min after start of nebulization. Additionally, a transit compartment was included for 5F-MDMB-P7AICA after inhalative administration in analogy to the present study. Taken together, the TK properties of structurally modified SCs with a carboxamide moiety appear to be very similar. However, in contrast to *cumyl*-5F-P7AICA, a four-compartment model describes the data for 5F-MDMB-P7AICA best.

Only limited data on fatal and non-fatal human intoxications involving the SC *cumyl*-5F-P7AICA have already been reported (Halter et al. 2019; Kleis et al. 2020; Zawadzki et al. 2021). These cases have in common that additional SCs or exogenous substances were found besides *cumyl*-5F-P7AICA. This finding could at least in parts be anticipated due to the manufacturing process of such herbal mixtures, which often contain various different SCs in one package. Consequently, the specific SC composition and quantity remain unknown for the consumer potentially leading to mixed intoxications. Halter et al. published data on several intoxications primarily associated with the consumption of the SC *cumyl*-PEGACLONE (Halter et al. 2019). In three cases, *cumyl*-5F-P7AICA was additionally detected with concentrations of 2.5 ng/mL and approximately 0.03 ng/mL in serum, as well as 0.23 ng/mL in femoral blood. Furthermore, a series of several (fatal) intoxications after consumption of the SC 5F-MDMB-PICA included one fatality related to a combination of various other SCs and *cumyl*-5F-P7AICA with < 0.1 ng/mL of *cumyl*-5F-P7AICA detected in femoral blood (Kleis et al. 2020). To date, only one fatal mono intoxication with *cumyl*-5F-P7AICA has been reported (Zawadzki et al. 2021). Upon the toxicological examination, 2.8 and 3.1 ng/mL of *cumyl*-5F-P7AICA were found in blood and urine specimens, respectively. Taken together, the data already published up to now correspond well with the data obtained in the current TK study, suggesting in non-fatal intoxications that *cumyl*-5F-P7AICA had been consumed a considerable time before blood sampling. However, several issues must be taken into account. For one, the ingested dose of *cumyl*-5F-P7AICA remains unclear in these cases and a more recent consumption of a lower dose might also be conceivable. Furthermore, the route of consumption (inhalative vs. oral) must be taken into account, resulting in possibly lower substance concentration in vivo. As SCs are commonly smoked by consumers in the form of a joint, a heat-related pyrolytic degradation must be considered, as already reported for other SCs (Kaizaki-Mitsumoto et al. 2017; Franz et al. 2017). Whilst *cumyl*-5F-P7AICA was administered without any heat treatment inhalatively to pigs in the present study, a possible heat-related pyrolytic degradation might have led to lower serum concentrations in the context of human (fatal) intoxications. Regarding *cumyl*-5F-P7AICA, respective data are lacking. Nevertheless, this mechanism should be kept in mind when interpreting toxicological findings. On the other hand, some blood specimens were collected postmortem. In cases of a prolonged agony, further metabolism of the consumed substance might have occurred, leading to lower substance levels at the time of death as compared to C_max_. Finally, the postmortem interval is often unknown and a possible instability of *cumyl*-5F-P7AICA must be considered. Therefore, interpretation of such postmortem analytical results using the TK data obtained in the present study should be regarded with caution.

### Population TK model

A TK model was developed describing *cumyl*-5F-P7AICA and its metabolite NPA. Overall, parameters derived from the modeling analysis (Table 2) are comparable with those previously identified for JWH-210 and RCS-4 (Schaefer et al. 2016, 2018). Regarding the volume of distribution (V_ss_) calculated in the present study for *cumyl*-5F-P7AICA (9.2 L/kg^0.75^), similar values were reported by Schaefer et al. for JWH-210 and RCS-4 (4.91 and 15.97 L/kg^0.75^, respectively). Comparison of these results shows that V_ss_ of *cumyl*-5F-P7AICA lay between the values of JWH-210 and RCS-4 and therefore correspond very well with those of the ‘older’ SCs. Furthermore, the calculated half-lifes of 2.6 min (t_1/2α_), 32 min (t_1/2β_), and 146 min (t_1/2γ_) for *cumyl*-5F-P7AICA are also in agreement with those reported for JWH-210 and RCS-4 (t_1/2α_: 1.2 and 1.8 min, t_1/2β_: 8.9 and 11.3 min, t_1/2γ_: 160 and 162 min, respectively). This finding indicates that in vivo, no relevant cleavage of the incorporated carboxamide moiety in the chemical structure of *cumyl*-5F-P7AICA occurs. This is also consistent with the in vitro results reported by Wagmann et al. studying the stability of various SCs using human carboxylesterases (Wagmann et al. 2022). Furthermore, this finding is also consistent with a previous controlled in vivo pig study of the authors. In this study, no metabolite with a cleaved carboxamide moiety was detected in pig urine specimens (Walle et al. 2021).

Interestingly, to accurately describe the concentration of the NPA metabolite, transit compartments were necessary to account for the delay between the appearance of *cumyl*-5F-P7AICA and NPA in serum. Such a delay is typically not reported in TK modeling analyses involving metabolites. However, the formation of NPA from *cumyl*-5F-P7AICA requires several intermediate metabolism stages (Walle et al. 2021), which potentially explains its delayed appearance. Additionally, the sampling schedule of metabolites in TK studies is usually not as dense within the first min after dose administration as in our study. This might have prevented other analyses from identifying the time frame of metabolite formation within the first min. Interestingly, this delay differed between inhalative and i.v. application and amounted to 12 and 6.4 min. Additionally, the fraction metabolized differed with 12 and 24%, following inhalative and i.v. application, respectively. However, the origin of these differences remains unknown.

To sum up, the TK data obtained in the current study are in a rather good agreement with those reported in literature for several (fatal) intoxications. In comparison to the TK properties of JWH-210 and RCS-4, no substantial differences were detected for *cumyl*-5F-P7AICA. Therefore, structural modifications of SCs with the incorporation of a carboxamide moiety as a linker between the core structure and the bridge residue do not appear to have a relevant influence on the TK properties.

## Conclusion

Based on concentration–time profiles of *cumyl*-5F-P7AICA and its NPA metabolite in pig serum, a popTK model was successfully developed. A three-compartment model with linear clearance describes best the pig serum concentration–time profiles of *cumyl*-5F-P7AICA, while a two-compartment model was better for the NPA metabolite. Following, the final popTK model was used for prediction of the human exposure of *cumyl*-5F-P7AICA and its NPA metabolite by allometric scaling. Compared to ‘older’ SCs (e.g. RCS-4 and JWH-210), which do not contain a carboxamide moiety, similar TK data were determined for *cumyl*-5F-P7AICA in the present study. Therefore, the incorporation of a carboxamide moiety as a linker is a modification in the chemical structure of SCs that does not result in any relevant changes in the TK properties.

## Supplementary Information

Below is the link to the electronic supplementary material.

Supplementary file1 (DOCX 204 KB)

Supplementary file2 (PPTX 83 KB)

Supplementary file3 (PPTX 253 KB)

Supplementary file4 (PPTX 514 KB)

## Data Availability

All data generated or analyzed during this study are included in this published article and its supplementary information files.
