# Supplementary material for: Does a carboxamide moiety alter the toxicokinetics of synthetic cannabinoids? A study after pulmonary and intravenous administration of cumyl-5F-P7AICA to pigs
Source: Arch Toxicol. 2024 Dec 4;99(2):633–43. doi: 10.1007/s00204-024-03906-z (PMC11775056; doi:10.1007/s00204-024-03906-z)
Supplement: Supplementary file 1 — Supplementary file1 (DOCX 204 KB) [file 204_2024_3906_MOESM1_ESM.docx]

Archives of Toxicology

**Supplementary Information**

**Does a carboxamide moiety alter the toxicokinetics of synthetic cannabinoids? - A study after pulmonary administration of cumyl-5F-P7AICA to pigs**

**Nadja Walle^1^, Christiane Dings^2^, Omar Zaher^2^, Adrian A. Doerr^1^, Benjamin Peters^1^, Matthias W. Laschke^3^, Thorsten Lehr^2^, Michael D. Menger^3^, Peter H. Schmidt^1^, Markus R. Meyer^4^ and Nadine Schaefer^1*^**

**___________________________________________________________________________**

^*^Correspondence to: Nadine Schaefer, Institute of Legal Medicine, Saarland University,

Building 49.1, 66421 Homburg, Germany

Email: [nadine.schaefer@uks.eu](mailto:nadine.schaefer@uks.eu)

^1^Institute of Legal Medicine, Saarland University, Building 49.1, 66421 Homburg, Germany

^2^Department of Clinical Pharmacy, Saarland University, Building C5 3, 66123 Saarbruecken, Germany

^3^Institute for Clinical & Experimental Surgery, Saarland University, Building 65/66, 66421 Homburg, Germany

^4^Department of Experimental and Clinical Toxicology, Center for Molecular Signaling (PZMS), Saarland University, Building 46, 66421 Homburg, Germany

**Materials and** **methods**

**Chemicals and reagents**

Ethylenediaminetetraacetic acid disodium salt (Na_2_EDTA), glacial acetic acid, and di-potassium hydrogen phosphate were purchased by Merck (Darmstadt, Germany). Acetonitrile (Optima), formic acid (Optima), water (Optima), ethanol (HPLC grade), methanol (HPLC grade), and acetone p.a. were bought from Fisher Scientific (Loughborough, United Kingdom). For drug administration, *cumyl*-5F-P7AICA (purity 99.72 %; Fig. 1A) was offered by the German Federal Criminal Police Office (Wiesbaden, Germany). *Cumyl*-5F-P7AICA (1 mg, solid) for validation, its *N*-pentanoic acid metabolite (NPA; 1 mg; solid), 5F-MDMB-P7AICA (1 mg; solid), the standards RCS-4-d_9_ (1 mg; solid) and AB-FUBINACA-d_4_ (1 mg; solid), as well as AB-005, AB-FUBINACA, AM-694, AM-697, AM-1220, AM-1248, AM-2201, AM-2232, AM2233, EAM-2201, JWH-015, JWH-081, JWH-122, JWH182, JWH-200, JWH-203, JWH-210, JWH-250, MAM2201, 5F-MDMB-PINACA, PB-22, 5F-PB-22, Pravadoline, RCS-8, RCS-4, UR-144 and XLR-11 were obtained from Cayman Europe (Tallinn, Estonia). Additionally, ∆^9^-tetrahydrocannabinol (THC), 11-hydroxy-THC (11-HO-THC), 11-nor-9-carboxy-THC (THC-COOH), cannabinol and cannabidiol were bought from Sigma-Aldrich (Munich, Germany), whilst JWH-018 was obtained by THC Pharm (Frankurt/Main, Germany).

## **Preparations**

**Buffer solution**

Preparation of the phosphate buffer (0.1 M, pH 9) was performed in accordance to previous studies (Schaefer et al. 2015, 2017, 2019, 2020; Doerr et al. 2024b; Walle et al. 2024a). Analogously, 22.82 g di-potassium hydrogen phosphate was dissolved in 1 L HPLC grade water.

**Blank whole blood and serum samples**

For method validation as well as the preparation of calibration standards and quality control (QC) samples, blank whole blood specimens were obtained from drug free pigs (Swabian Hall strain, Emil Faerber GmbH & Co. KG, Zweibruecken, Germany), divided into two aliquots, and centrifuged at 1,476  x *g* for 15 min to obtain serum specimens, as already published in previous studies (Schaefer et al. 2015; Nordmeier et al. 2021; Doerr et al. 2024a). To prevent coagulation, 1.64 mg/mL Na_2_EDTA were added to the whole blood samples. Following, serum and whole blood specimens were stored at - 20 °C.

**Stock solutions, calibration standards, and QC samples**

The solid substances *cumyl*-5F-P7AICA, its NPA metabolite, RCS-4-d_9_, and AB-FUBINACA-d_4_ were dissolved in ethanol to obtain standard stock solutions with conc. of 1000 µg/mL. Subsequently, the prepared standard stock solutions were dissolved with ethanol, to create working standard solutions containing 0.01 µg/mL, 0.1 µg/mL, 1 µg/mL, and 10 µg/mL of the respective substance. For preparation of the spiking solutions for calibration standards, the standard stock solutions were also diluted with ethanol, to receive final serum conc. of 0.1, 0.3, 0.5, 0.7, 0.9, and 1.1 ng/mL of the NPA metabolite and 0.5, 10, 20, 30, 40, and 50 ng/mL of *cumyl*-5F-P7AICA. The QC spiking solutions low and high, containing 0.2 ng/mL and 5 ng/mL or 0.8 ng/mL and 35 ng/mL of the NPA and *cumyl*-5F-P7AICA respectively, were prepared analogously. The prepared solutions were stored at – 20 °C.

**In vitro drug delivery efficiency test**

To assess the drug delivery efficiency of *cumyl*-5F-P7AICA after nebulization, an in vitro drug delivery efficiency test was performed in accordance to the setup already published by Schaefer et al. (Schaefer et al. 2018a) with a few modifications. In brief, 1 mL of a stock solution containing 0.5 mg/mL *cumyl*-5F-P7AICA was prepared by dissolving the solid substance in ethanol. Using the M-neb flow+ ventilation ultrasonic nebulizer MN-300/7 (Nebutec, Elsenfeld, Germany), the prepared solution was nebulized under ventilation applying the inspiration-triggered mode. The settings of the ventilation were in accordance to the in vivo pig studies (Schaefer et al. 2019; Walle et al. 2021; Doerr et al. 2021). After nebulization, the aerosol was transferred via the endotracheal tube, through a glass fiber filter (GFF) into a simulated pig lung (anesthesia bag). To ensure that only inhaled air and no exhaled breath can pass the GFF, two check valves have been installed in the experimental setup. The procedure was repeated six times. To assess the drug delivery efficiency, *cumyl*-5F-P7AICA was extracted from the components of the experimental setup in accordance to an already published study by Schaefer et al. (Schaefer et al. 2018a), with slight modifications. Therefore, GFF were initially macerated by adding 5 mL acetone. The solution was ultrasonicated for 10 min and centrifuged at 2,898 x *g* for 8 min. Subsequently, the solution obtained was filtrated using chromafil filters (1.0 µm pore size, Macherey-Nagel, Düren, Germany). Afterwards, the solution was diluted 1:50 using ethanol in a first step. Secondly, the obtained dilution was further diluted 1:20 with a mixture (50:50, *v/v*) of mobile phases A (0.1% aqueous formic acid) and B (0.1% formic acid in acetonitrile). After the addition of 25 µL of a stable isotope-labeled internal standard (SIL-IS; AB-FUBINACA-d4, 50 ng/20 µL), 20 µL of the extracts were analysed by liquid chromatography (LC)-tandem mass spectrometry (MS). In order to calculate the drug delivery efficiency, the measured values were adjusted by the extraction efficiency of 90% using acetone as it was found in the context of another study (Walle et al. 2024b)

To determine the drug residues in the different experimental compounds, such as the endotracheal tube, the heat and moisture exchanger (HME) filter, the reservoir of the nebulizer, and the anesthesia bag, the respective part of the set up (n=6 each) was cut into pieces, mixed with ethanol, and macerated via ultrasonication for 10 min, respectively. As far as the reservoir of the nebulizer is concerned, the components (n=6) were only rinsed with ethanol. Subsequently, 20 or 25 µL of the SIL-IS (50 ng/25 µL for the reservoir of the nebulizer, and 5 ng/20 µL for the remaining components) and 25 µL of the respective solution were diluted 1:20 with a mixture (50:50, *v/v*) of mobile phases A and B. Following, 20 µL of the solutions were measured by LC-MS/MS.

The amount of the substance contained were determined by comparing the ratios with those of a reference solution measured at the same time.

## **In vivo study**

**Surgical procedures**

The surgical procedures in the present work were in accordance to those already published in previous studies (Schaefer et al. 2019; Walle et al. 2021, 2024a; Doerr et al. 2021, 2024a, b; Nordmeier et al. 2022), with the modification, that 5 mg/kg body weight (BW) of carprofen (Rimadyl; Zoetis, Berlin, Germany) was additionally given to the pigs as a pain medication.

In brief, a mixed intramuscular injection of xylazine hydrochloride (2.5 mg/kg BW, Rompun^®^, Bayer, Leverkusen, Germany), ketamine hydrochloride (30 mg/kg BW, Ursotamin^®^; Serumwerke Bernburg, Bernburg, Germany), and atropine (1 mg, Braun, Melsungen, Germany) was administered to the animals for anesthetization. For sufficient fluid substitution (0.9% sodium chloride, 8 mL/kg/h, Braun, Melsungen, Germany), a 20G indwelling needle was inserted percutaneously in the left ear. The pigs were endotracheally intubated, mechanically ventilated with a volume-controlled ventilation with an oxygen and air mixture (1:2, *v/v*, FiO2 of 0.30, Respirator ABV-U; F. Stephan GmbH, Gackenbach, Germany) and a tidal volume of 10-12 mL/kg BW. Anesthesia was achieved by an inhalative administration of 2-4% isoflurane (Forene, AbbVie, Ludwigshafen, Germany). In addition, a triple-lumen 7F central venous catheter (Certofix Trio, Braun, Melsungen, Germany) was placed in the jugular vein for blood sampling, intravenous (i.v.) administration or monitoring of the mean central venous pressure. Additionally, a suprapubic bladder catheter (Cystofix, Braun, Melsungen, Germany) was placed in the bladder for continuous urine collection, and an arterial catheter (Leadercath Expert 14G, Vygon, Aachen, Germany) in the left formal artery for invasive blood pressure measurement. The animals were then allowed to stabilize for 10-15 min.

**Sample preparation**

For sample preparation, 500 µL serum or blood was added to 2.5 mL phosphate buffer (pH 9), 50 µL ethanol and 20 µL of the SIL-IS RCS-4-d_9_ (1 ng/20 µL) and AB-FUBINACA-d_4_ (0.25 ng/20 µL) each. After vortexing and centrifugation at 2,898 x *g* for 8 min, the solutions were loaded onto conditioned (2 x 3 mL methanol and 1 x 3 mL 0.1 M phosphate buffer pH 9) Strata C_18_ endcapped cartridges (200 mg/3 mL; Phenomenex LTD, Aschaffenburg, Germany) cartridges. After washing steps with 3 mL phosphate buffer, 3 mL acetic acid (0.25 M), and 3 mL water, 60 µL acetone were added and the cartridges were dried under a maximum vacuum of 10 inHg. Elution of the substances was carried out through the addition of 1.5 mL methanol-acetone (1:1, *v/v*) and the eluates were dried under a gentle stream of nitrogen at 60 °C. The dried residue was dissolved by adding 50 µL of a mixture (50:50, *v/v*) of mobile phases A and B. Afterwards, 5 µL were injected onto a LC quadrupole time-of-flight (TOF)- MS system.

**Method validation**

Method validation was performed in accordance to international guidelines (Peters et al. 2007) and the guidelines of the Society of Toxicological and Forensic Chemistry (Peters et al. 2009). Moreover, the procedure was already described in previous studies (Nordmeier et al. 2021; Walle et al. 2022). In this context, the analytical parameters selectivity, limits of detection (LODs), linearity, lower limits of quantification (LLOQs), recovery (RE), matrix effects (ME), process efficiency (PE), intra- and interday accuracy and precision tests, freeze/ thaw, long-term, and processed sample stability as well as carry-over effects were examined in order to achieve a full validation of the method for pig whole blood and serum samples.

First of all, selectivity of the method was checked. For this purpose, six blank pig serum or blood samples from different drug-free pigs were used. Additionally, two zero samples containing only the SIL-IS and one sample spiked with possible interfering substances (AB-005, AB-FUBINACA, AM-694, AM-697, AM1220, AM-1248, AM-2201, AM-2232, AM-2233, EAM2201, JWH-018, JWH-015, JWH-081, JWH-122, JWH-182, JWH-200, JWH-203, JWH-210, JWH-250, MAM-2201, 5F-MDMB-PINACA, PB-22, 5F-PB-22, Pravadoline, RCS-8, RCS-4, UR-144, XLR-11, THC, 11-THC-OH, THC-COOH, cannabidiol, cannabinol, and 5F-MDMB-P7AICA) in final concentrations (conc.) of 100 ng/mL each were prepared. After analyzing, the samples were checked for possible interfering signals at the multiple reaction monitoring (MRM) transitions of the SIL-IS, *cumyl*-5F-P7AICA and the NPA, respectively.

Following, LODs were examined. Therefore, blank pig serum and blood samples were spiked with different conc. of *cumyl*-5F-P7AICA and the NPA metabolite. The final blood and serum conc. of the substances investigated were as follows: 0.05, 0.1, 0.15, 0.2, 0.25, 0.3, 0.35, 0.4, 0.45, and 0.5 ng/mL as well as 0.001, 0.0025, 0.005, 0.01, 0.015, 0.02, 0.025, 0.03, 0.035, 0.04 ng/mL for the parent compound and the metabolite, respectively. Determination of the LODs was carried out by verifying the respective signal-to-noise ratios of the qualifier and quantifier MRM transitions (> 3:1).

For checking the linearity of the calibration, 500 µL blank pig blood or serum specimens were spiked with 25 µL of six different calibration standard conc., respectively. Final blood/ serum calibration standard conc. were: 0.5, 10, 20, 30, 40, 50 ng/mL and 0.1, 0.3, 0.5, 0.7, 0.9, and 1.1 ng/mL for *cumyl*-5F-P7AICA and its metabolite respectively. Each calibrator was analyzed under repeated conditions six times. For evaluation, the peak area ratios (analyte/ SIL-IS) were calculated and plotted against the respective calibrator conc. Valistat 2.0 software (Arvecon, Walldorf, Germany) was used for linear regression applying a non-weighted, weighted [1/conc.], and a weighted least-square [1/(conc.)²] regression model was applied. Moreover, a Grubbs test, F test, and a Mandel test were performed. The LLOQ were set at the lowest calibrator in serum or blood, respectively.

To investigate the accuracy and precision of the method, 500 µL blank pig blood or serum were freshly extracted in duplicates on 8 different days using the standard extraction procedure. Before, 25 µL of each QC low or high solution, with final conc. of *cumyl*-5F-P7AICA or the NPA metabolite of 5 and 0.2 ng/mL or 35 and 0.8 ng/mL respectively, were added. The corresponding conc. of the QCs were finally determined by means of a daily calibration curve. For calculation of intermediate precision, repeatability, and bias values, Valistat 2.0 software was used.

To examine RE, ME, and PE, three different approaches were prepared in accordance to Matuszewski et al. applying drug-free pig blood or serum from five different pigs (Matuszewski et al. 2003). In this context, set 1 (spiked matrix samples) represents the standard sample preparation in which 25 µL of each QC low or high spiking solution of *cumyl*-5F-P7AICA and the NPA metabolite to 500 µL blank blood or serum before solid phase extraction (SPE). For preparation of set 2 (spiked extracts), the respective QC solutions were only added after the extraction procedure. Set 3 (control samples) represented the standard QC spiking solution without any extraction procedure or the addition of blank pig blood or serum, respectively. For evaluation of the ME, the absolute peak areas of the spiked extracts (set 2) were set into relation to those received after analyzing the control samples (set 3). Additionally, RE was evaluated by comparing the absolute peak areas of the spiked matrix samples (set 1) with those of the spiked extracts (set 2), whilst PE was obtained by comparing the results of the spiked matrix samples (set 1) with the control samples (set 3).

Regarding the stability of the substances in the matrices examined, various approaches were carried out. First, the freeze-thaw stability was investigated. Therefore, 500 µL blank pig blood or serum was spiked with 25 µL of the respective QC low or high spiking solution, whereby a sixfold preparation was carried out for each QC level. Following, extraction and analysis of the respective samples was conducted after three freeze/ thaw cycles, with each cycle consisting of at least 20 h of freezing at – 20 °C with a following thawing of about 1 h at room temperature. The long-term stability was also examined by spiking 500 µL blank pig blood or serum with 25 µL of the respective QC low or high spiking solution (n=6 each). In contrast to the freeze-thaw stability, analysis of the samples was carried out 14 days after freezing at – 20 °C. To interpret the results, an additional daily extraction of six blank pig blood or serum samples was performed after the addition of 25 µL QC low or high, respectively. The remaining amount of the analytes in the treated matrices were calculated by means of a daily calibration curve. Furthermore, to investigate the processed sample stability, 500 µL blank pig blood or serum was spiked with 25 µL of the respective QC low or high spiking solution (n=6 each) and extracted by SPE. After the addition of a mixture (50:50, *v/v*) of mobile phases A and B, the respective solutions were pooled and six-fold aliquoted. Considering, that analysis of an authentic LC-TOF-MS sequence takes at least about 6 h, the prepared samples were evenly distributed over time, with time intervals of about 80 min. For calculation of the processed sample stability, the absolute peak areas of the respective analyte were plotted against the time of injection, and linear regression was performed using Valistat 2.0.

Finally, the carry over effects were checked during an authentic series of measurements, whilst one blank sample was analyzed between two authentic samples, containing high analyte conc.

**LC-MS/MS apparatus**

**LC-triple-quadrupole MS**

In accordance to previous studies (Schaefer et al. 2018a; Walle et al. 2022, 2024a), a high pressure LC system (Thermo Fisher, Dreieich, Germany) was used for analyses of the drug delivery efficiency test. The system was composed of one Allegro pump and an HTC PAL autosampler. For chromatographic separation, the system was equipped with a Gravity C18 column (150x2 mm, 5 µm, Macherey-Nagel, Dueren, Germany) and gradient elution was carried out using mobile phases A and B. Therefore, the system started with 25% mobile phase B (flow rate 0.5 mL/min). This setting was kept for 1 min. Following, the amount of mobile phase B increased within 4 min to 100% and kept for 4 min. Finally, 25% mobile phase B were recovered and hold for 1 min. The total run time was set at about 10 min.

The LC system used was linked to a TF TSQ Quantum Ultra Accurate Mass triple stage MS, equipped with an electrospray ionization (ESI) interface. The source was set in positive mode. The settings of the MS were as follows: capillary temperature, 290 °C; vaporizer temperature, 380 °C; sheath gas, 40 arbitrary units (AU); auxiliary gas, 20 AU; ion sweep gas, 5 AU; spray voltage, 4.000 V; collision cell pressure, 1.5 mTorr. The MRM mode with three transitions per precursor ion was used for the detection of the substances and TF Xcalibur Version 2.0.7 SP 1 software was used for evaluation.

**LC-TOF-MS**

In accordance to previous studies (Walle et al. 2022, 2024a), a SCIEX TripleTOF 6600+ system (AB SCIEX, Concord, USA) was used for method validation and analysis of pig blood and serum samples. The system was connected to an Exion LC system (AB SCIEX), consisting of 2 AD pumps, an AD column oven as well as an AD autosampler. For chromatography a nucleoshell PFP column (100x2 mm, 2.7 µm; Macherey-Nagel, Dueren, Germany) was used. The column oven temperature has been 40 °C, the total runtime was set to 10 min with the following gradient elution settings: start with 25% mobile phase B with a flow rate of 0.5 mL/min and kept for 1 min. After a following increase to 99% mobile phase B over 3 min and holding for 3 min, the starting conditions were restored and hold for 2 min.

The instrumentation and settings of the MS were also according to the aforementioned studies. Analogously, the MS was equipped with a DuoSpray source running in positive ESI mode. The settings were: collision energy spread, 15 V; collision energy, 35 V; ion spray voltage, 4.500 V; declustering potential, 80 V; capillary temperature, 450 °C; ion source gas 1, 45 AU; ion source gas 2, 57 AU; curtain gas, 30 AU. After 5 runs, the system was mass calibrated using a calibrant delivery system. For detection and quantification of *cumyl*-5F-P7AICA and its NPA metabolite, the product ion scan mode was used. Sciex OS version 1.6.1.29803 software was used for evaluation.

**Results**

**In vitro drug delivery efficiency**

**Drug delivery efficiency**

To determine the drug delivery efficiency of *cumyl*-5F-P7AICA, the ratios of the nebulized drug were compared to those of the respective extracted drug using acetone as the extraction solvent, as already found in the context of a previous work (Walle et al. 2024b). This resulted in a drug delivery efficiency of 74 ± 10% for *cumyl*-5F-P7AICA.

**Drug residues in the different components of the experimental setup**

A small portion of approx. 10.3 ± 3.0% of the initial nebulized dose of *cumyl*-5F-P7AICA could be found in the remaining components of the experimental setup by separate refurbishment and analysis. Specifically, an amount of 6.3 ± 2.2%, 2.5 ± 0.54%, and 1.5 ± 0.23% of the drug could be found in the reservoir of the nebulizer, the endotracheal tube, and the HME filter (n=6 each), respectively. Analysis of the anesthesia bag (n=6) yielded negative results.

**Method development and validation**

In the present work, a SPE was performed in accordance to the method published by Schaefer et al. for other SCs (Schaefer et al. 2015, 2016, 2018b). However, slight modifications were made. First, 20 µL RCS-4-d_9_ and AB-FUBINACA-d_4_ each were used as SIL-IS for quantification of *cumyl*-5F-P7AICA and the NPA, respectively. In addition, a larger amount of ethanol (50 µL) was added. Moreover, after evaporation under nitrogen, the dry residues were dissolved in 50 µL of a mixture of mobile phases A and B (50:50, *v/v*) instead of 100 µL. The following analysis was performed using a LC-quadrupole TOF-MS system.

First of all, it had to be noted, that all of the validation parameters tested in the present work were in the specified range as recommended in the guidelines of the GTFCh (Peters et al. 2009). Regarding the selectivity of the method, no interfering signals were detected at the MRM transitions of the examined compounds when analyzing blank pig blood and serum or zero samples. Additionally, no similar MRM could be observed by the spiked substances.

The LOD was set at 0.06 ng/mL and 0.07 ng/mL for *cumyl*-5F-P7AICA as well as 0.006 ng/mL and 0.005 ng/mL for the NPA metabolite in blood and serum samples, respectively.

As far as the linearity of the calibration is concerned, a linear calibration was used with weighting factors of 1/x² for the metabolite and 1/x for the parent substance. In both matrices examined, the calibration range was set to 0.1 – 1.1 ng/mL and 0.5 – 50 ng/mL for the metabolite and *cumyl*-5F-P7AICA, respectively. In addition, the lowest calibrator each was defined as LLOQ.

Regarding the accuracy and precision of the method, the bias values with the respective relative standard deviation (RSD) as well as the intermediate precision RSD for QC low and high in blood and serum samples are shown in Table SI 1. Regarding these data, accuracy and precision of the method is given, as the values fulfilled the criteria of the GTFCh guidelines pretending, that the bias values should be lay within 15% of the nominal conc. and the RSD values should be ≤15% (Peters et al. 2009).

The RE, ME, and PE values for QC low and high in blank pig blood and serum samples are depicted in Table SI 1. As can be seen here, the ME variability is < 25%, so that the requirements of the GTFCh guidelines are also complied here (Peters et al. 2009).

As far as the stability of the tested compounds is concerned, the freeze-thaw and long-term stability were initially tested. In both approaches, no relevant decrease in conc. of *cumyl*-5F-P7AICA and its NPA metabolite could be detected. Therefore, the validation criteria which say, that the mean conc. of the analyte after treatment has to be within ±10% of the control samples and the 90% confidence interval has to be in the range of ±20% of the control samples, was met. Subsequently, the processed sample stability of both analytes was tested at about 5 °C over 6h. Here, no relevant decrease in peak area could be detected over the time of an authentic sequence, showing, that an instability of *cumyl*-5F-P7AICA and its metabolite has not to be expected during this time.

Finally, no carry-over effects were detected.

| **Table SI 1** Limit of detection (LOD), calibration range, nominal concentration (conc.), accuracy, repeatability, intermediate precision as well as results of matrix effect (ME), recovery (RE), and process efficiency (PE) studies of quality control (QC) low and high samples containing *cumyl*-5F-P7AICA or its *N*-pentanoic acid metabolite (NPA) in pig serum and blood. | | | | |
| --- | --- | --- | --- | --- |
| **Validation**  **parameter** | ***Cumyl*-5F-P7AICA** | | **NPA** | |
|  | **Serum** | **Blood** | **Serum** | **Blood** |
| LOD [ng/mL] | 0.07 | 0.06 | 0.005 | 0.006 |
| Calibration range [ng/mL] | 0.5 - 50 | 0.5 - 50 | 0.1 – 1.1 | 0.1 – 1.1 |
| **QC low** | | | | |
| Nominal conc. [ng/mL] | 5 | 5 | 0.2 | 0.2 |
| Accuracy bias [%] | 11 | -7.4 | 3.0 | -2.1 |
| Repeatability RSD [%] | 2.9 | 3.1 | 3.3 | 6.3 |
| Intermediate Precision RSD [%] | 4.8 | 5.0 | 6.2 | 6.3 |
| ME % (RSD %) | 78 (7.2) | 76 (9.9) | 86 (12) | 88 (9.7) |
| RE % (RSD %) | 90 (4.9) | 86 (5.7) | 92 (5.2) | 87 (11) |
| PE % (RSD %) | 71 (8.4) | 66 (7.6) | 79 (10) | 77 (13) |
| **QC high** | | | | |
| Nominal conc. [ng/mL] | 35 | 35 | 0.8 | 0.8 |
| Accuracy bias [%] | 3.4 | 3.5 | 3.1 | -1.3 |
| Repeatability RSD [%] | 4.9 | 6.8 | 3.3 | 5.0 |
| Intermediate Precision RSD [%] | 4.9 | 7.0 | 5.8 | 6.1 |
| ME % (RSD %) | 87 (7.3) | 80 (5.0) | 81 (10) | 76 (5.2) |
| RE % (RSD %) | 85 (4.8) | 84 (3.1) | 85 (6.8) | 91 (5.6) |
| PE % (RSD %) | 74 (4.1) | 68 (6.1) | 69 (5.7) | 69 (8.3) |

**Discussion**

**In vitro drug delivery efficiency**

Regarding the results of the in vitro drug delivery efficiency test, 74 ± 10% of the nebulized SC dose was found in the glass fiber filters, reflecting the deposition of the substance in the pig lung. Furthermore, a total amount of approx. 10.3 ± 3.0% of the initial nebulized SC dose was found in the remaining experimental components. Consequently, a loss of approx. 15% of the nebulized dose is not comprehensible.

If one has a look to the results published by Schafer et al for JWH-210, RCS-4 and THC (Schaefer et al. 2018a), a drug delivery efficiency of 70.5 – 78.8% of the nebulized dose was reported. Furthermore, approx. 10% of the nebulized dose were found by Schaefer et al in the nebulizer device and the endotracheal tube, resulting in an inexplicable loss of substance of about 10-20% (Schaefer et al. 2018a). Comparing these results with those obtained in the present work it can be noticed that similar findings were observed for *cumyl*-5F-P7AICA. However, compared to the herein published data, a slightly higher amount of the SCs was found in the endotracheal tube by Schaefer et al (Schaefer et al. 2018a). As it is already known, (synthetic) cannabinoids have a high tendency to affect to plastic surfaces due to their lipophilicity (Christophersen 1986; Kneisel et al. 2013). Hence, a possible explanation for the slightly higher amount of SCs deposition in the endotracheal tube reported by Schaefer et al as compared to the findings presented here might be in the lower lipophilicity of *cumyl*-5F-P7AICA (LogP = 4.20) as compared to JWH-210 (LogP = 7.5) or RCS-4 (LogP = 5.6) (Schaefer et al. 2019). However, analysis of the HME filter and the anesthesia bag were not carried out by Schaefer et al.

Overall, the findings of the present study for *cumyl*-5F-P7AICA are in good agreement to those published by Schaefer et al for JWH-210, RCS-4, and THC.

**Final Model**

**Differential equations**

Inhalation absorption and dosing compartment:

DADT(1) = -ka*A(1)

Central compartment *cumyl*-5F-P7AICA:

DADT(2) = ka*A(2) – CL_i_/V_i_*A(2) – Q1_i_/V_i_*A(2) + Q1_i_/V_P1i_*A(4) – Q2_i_/V_i_*A(2) + Q2_i_/V_P2i_*A(5)

Central compartment NPA:

DADT(3) = ktr*(A(7)*AP + A(8)*(1-AP)) – CL_Mi_/V_M_*A(3) – QM_i_/V_Mi_*A(3) + QM_i_/V_PMi_*A(6)

1^st^ peripheral compartment *cumyl*-5F-P7AICA:

DADT(4) = Q1_i_/V_i_*A(2) – Q1_i_/V_P1i_*A(4)

2^nd^ peripheral compartment *cumyl*-5F-P7AICA:

DADT(5) = Q2_i_/V_i_*A(2) – Q2_i_/V_P2i_*A(5)

1^st^ peripheral compartment NPA:

DADT(6) = QM_i_/V_Mi_*A(3) – QM_i_/V_PMi_*A(6)

Metabolism delay compartment 1:

DADT(7) = CL_i_/V_i_*A(2)*f_MET_*(1+AP*f_APP_)*F_WGT – ktr*A(7)

Metabolism delay compartment 2:

DADT(8) = ktr*A(7) – ktr*A(8)

**Parameter calculation**

AP = 0 for inhalation and 1 for iv application

WGT = individual weight of the pig

CL_i_ = CL* WGT**0.75

V_i_ = V2* WGT**0.75

Q1_i_ = Q1* WGT**0.75

V_P1i_ = V_P1_* WGT**0.75

Q2_i_ = Q2 * WGT**0.75

V_P2i_ = V_P2_* WGT**0.75

CL_Mi_ = CL_M_ * WGT**0.75

QM_i_ = QM * WGT**0.75

V_PMi_ = V_PM_ * WGT**0.75

V_Mi_ = V_i_

WGT_Parent = 367.5 🡪 Molecular weight parent

WGT_Metabolite = 379.5 🡪 Molecular weight metabolite

F_WGT = WGT_Metabolite/WGT_Parent

Concentration *cumyl*-5F-P7AICA: A(2)/V

Concentration NPA: A(3)/VM

**Referenzen**

Christophersen AS (1986) Tetrahydrocannabinol stability in whole blood: plastic versus glass containers. J Anal Toxicol 10(4):129-31. <https://doi.org/10.1093/jat/10.4.129>

Doerr AA, Nordmeier F, Walle N et al (2021) Can a recently developed pig model be used for in vivo metabolism studies of 7-azaindole-derived synthetic cannabinoids? A study using 5F-MDMB-P7AICA. J Anal Toxicol 45(6):593-604. <https://doi.org/10.1093/jat/bkaa122>

Doerr AA, Dings C, Zaher O et al (2024a) Toxicokinetic modelling of the synthetic cannabinoid 5F-MDMB-P7AICA and its main metabolite in pigs following pulmonary administration. Br J Clin Pharmacol, submitted.

Doerr AA, Nordmeier F, Walle N et al (2024b) Does a postmortem redistribution affect the concentrations of the 7 azaindole-derived synthetic cannabinoid 5F-MDMB-P7AICA in tissues and body fluids following pulmonary administration to pigs? Arch Toxicol 98(10):3289-3298. <https://doi.org/10.1007/s00204-024-03815-1>

Kneisel S, Speck M, Moosmann B, Auwaerter V (2013) Stability of 11 prevalent synthetic cannabinoids in authentic neat oral fluid samples: glass versus polypropylene containers at different temperatures. Drug Test Anal 5(7):602-6. <https://doi.org/10.1002/dta.1497>

Matuszewski BK, Constanzer ML, Chavez-Eng CM (2003) Strategies for the assessment of matrix effect in quantitative bioanalytical methods based on HPLC-MS/MS. Anal Chem 75(13):3019-30. <https://doi.org/10.1021/ac020361s>

Nordmeier F, Sihinevich I, Doerr AA et al (2021) Toxicokinetics of U-47700, tramadol, and their main metabolites in pigs following intravenous administration: is a multiple species allometric scaling approach useful for the extrapolation of toxicokinetic parameters to humans? Arch Toxicol 95(12):3681-3693. <https://doi.org/10.1007/s00204-021-03169-y>

Nordmeier F, Doerr AA, Potente S et al (2022a) Perimortem distribution of U-47700, tramadol and their main metabolites in pigs following intravenous administration. J Anal Toxicol 46(5):479-486. <https://doi.org/10.1093/jat/bkab044>

Peters FT, Drummer OH, Musshoff F (2007) Validation of new methods. Forensic Sci Int 165(2-3):216-24. <https://doi.org/10.1016/j.forsciint.2006.05.021>

Peters FT, Hartung M, Herbold M et al (2009) Anhang B zur Richtlinie der GTFCh zur Qualitätssicherung bei forensisch-toxikologischen Untersuchungen – Anforderungen an die Validierung von Analysenmethoden. Toxichem Krimtech 76, 185–208. <https://www.gtfch.org/cms/images/stories/files/GTFCh_Richtlinie_Anhang%20B_Validierung_Version%201.pdf>. Accessed Oktober 13, 2024.

Schaefer N, Kettner M, Laschke MW et al (2015) Simultaneous LC-MS/MS determination of JWH-210, RCS-4, ∆9-tetrahydrocannabinol, and their main metabolites in pig and human serum, whole blood, and urine for comparing pharmacokinetic data. Anal Bioanal Chem 407(13):3775-86. <https://doi.org/10.1007/s00216-015-8605-6>

Schaefer N, Wojtyniak J-G, Kettner M et al (2016) Pharmacokinetics of (synthetic) cannabinoids in pigs and their relevance for clinical and forensic toxicology. Toxicol Lett 253:7-16. <https://doi.org/10.1016/j.toxlet.2016.04.021>

Schaefer N, Kettner M, Laschke MW et al (2017) Distribution of synthetic cannabinoids JWH-210, RCS-4 and Δ 9-tetrahydrocannabinol after intravenous administration to pigs. Curr Neuropharmacol 15(5):713-723. <https://doi.org/10.2174/1570159X15666161111114214>

Schaefer N, Kroell AK, Laschke MW et al (2018a) Development of an in-vitro drug delivery efficiency test for a pulmonary toxicokinetic pig study. Curr Drug Deliv 15(8):1167-1171. <https://doi.org/10.2174/1567201815666180214130014>

Schaefer N, Wojtyniak J-G, Kroell AK et al (2018b) Can toxicokinetics of (synthetic) cannabinoids in pigs after pulmonary administration be upscaled to humans by allometric techniques? Biochem Pharmacol 155:403-418. <https://doi.org/10.1016/j.bcp.2018.07.029>

Schaefer N, Kroell AK, Koerbel C et al (2019) Distribution of the (synthetic) cannabinoids JWH-210, RCS-4, as well as ∆9-tetrahydrocannabinol following pulmonary administration to pigs. Arch Toxicol 93(8):2211-2218. <https://doi.org/10.1007/s00204-019-02493-8>

Schaefer N, Kroell AK, Koerbel C et al (2020) Time- and temperature-dependent postmortem concentration changes of the (synthetic) cannabinoids JWH-210, RCS-4, as well as ∆9-tetrahydrocannabinol following pulmonary administration to pigs. Arch Toxicol 94(5):1585-1599. <https://doi.org/10.1007/s00204-020-02707-4>

Walle N, Nordmeier F, Doerr AA, et al (2021) Comparison of in vitro and in vivo models for the elucidation of metabolic patterns of 7-azaindole-derived synthetic cannabinoids exemplified using cumyl-5F-P7AICA. Drug Test Anal 13(1):74-90. <https://doi.org/10.1002/dta.2899>

Walle N, Doerr AA, Laschke MW et al (2022) Systematic studies on temperature-dependent in vitro stability during storage and smoking of the synthetic cannabinoid 5F-MDMB-P7AICA. J Anal Toxicol 46(4):374-382. <https://doi.org/10.1093/jat/bkab022>

Walle N, Doerr AA, Peters B et al (2024a) Are the postmortem concentration changes of the synthetic cannabinoid cumyl-5F-P7AICA and its N-pentanoic acid metabolite dependent on the environmental conditions? – A systematic study following pulmonary administration to pigs. Toxicol Lett S0378-4274(24)02040-X. <https://doi.org/10.1016/j.toxlet.2024.10.006>

Walle N, Doerr AA, Peters B et al (2024b) Development and method validation of a sampling technique for a reproducible detection of synthetic cannabinoids in exhaled breath using an in vitro pig lung model. J Anal Toxicol bkae078. <https://doi.org/10.1093/jat/bkae078>

**Legend to figures**

**Fig. SI 1** Schematic representation of the final model structure. For intravenous application, only one transit compartment is used. For pulmonary application, two transit compartments are used.

**Fig. SI 2** Goodness-of-fit plots stratified by analyte and application. Upper row: Observations vs. population model predictions, lower row: Observations vs. individual model predictions.

**Fig. SI 3** Individual concentration-time profiles and model predictions. Points represent observations, dashed lines represent population predictions and full lines represent individual predictions.
