# Supplementary figures and images for: Does a carboxamide moiety alter the toxicokinetics of synthetic cannabinoids? A study after pulmonary and intravenous administration of cumyl-5F-P7AICA to pigs
Source: Arch Toxicol. 2024 Dec 4;99(2):633–43. doi: 10.1007/s00204-024-03906-z (PMC11775056; doi:10.1007/s00204-024-03906-z)

## Slide 1
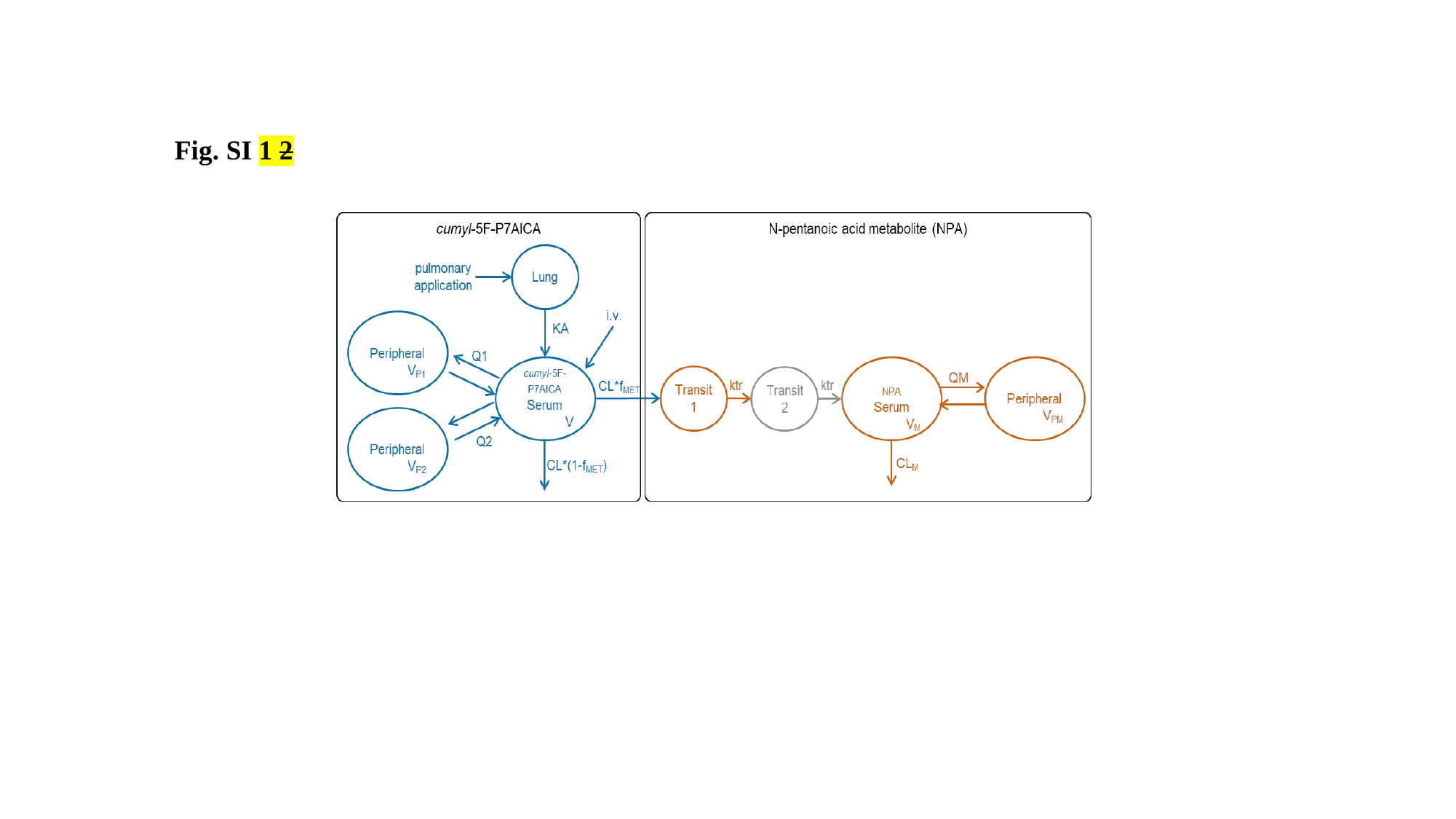

Fig. SI 1 2

Supplement: Supplementary file 2 — Supplementary file2 (PPTX 83 KB) [file 204_2024_3906_MOESM2_ESM.pptx]

## Slide 1
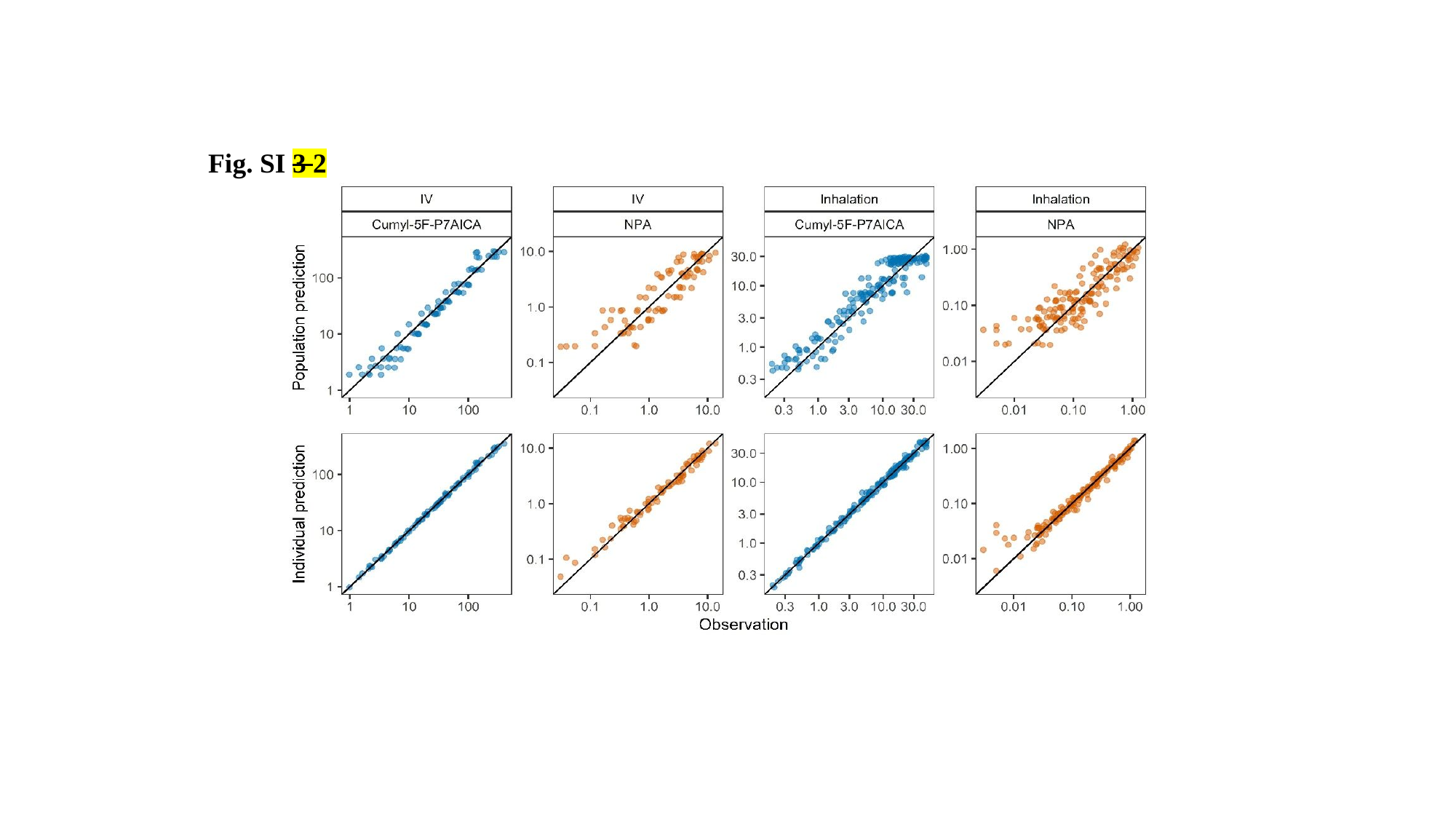

Fig. SI 3 2

Supplement: Supplementary file 3 — Supplementary file3 (PPTX 253 KB) [file 204_2024_3906_MOESM3_ESM.pptx]

## Slide 1
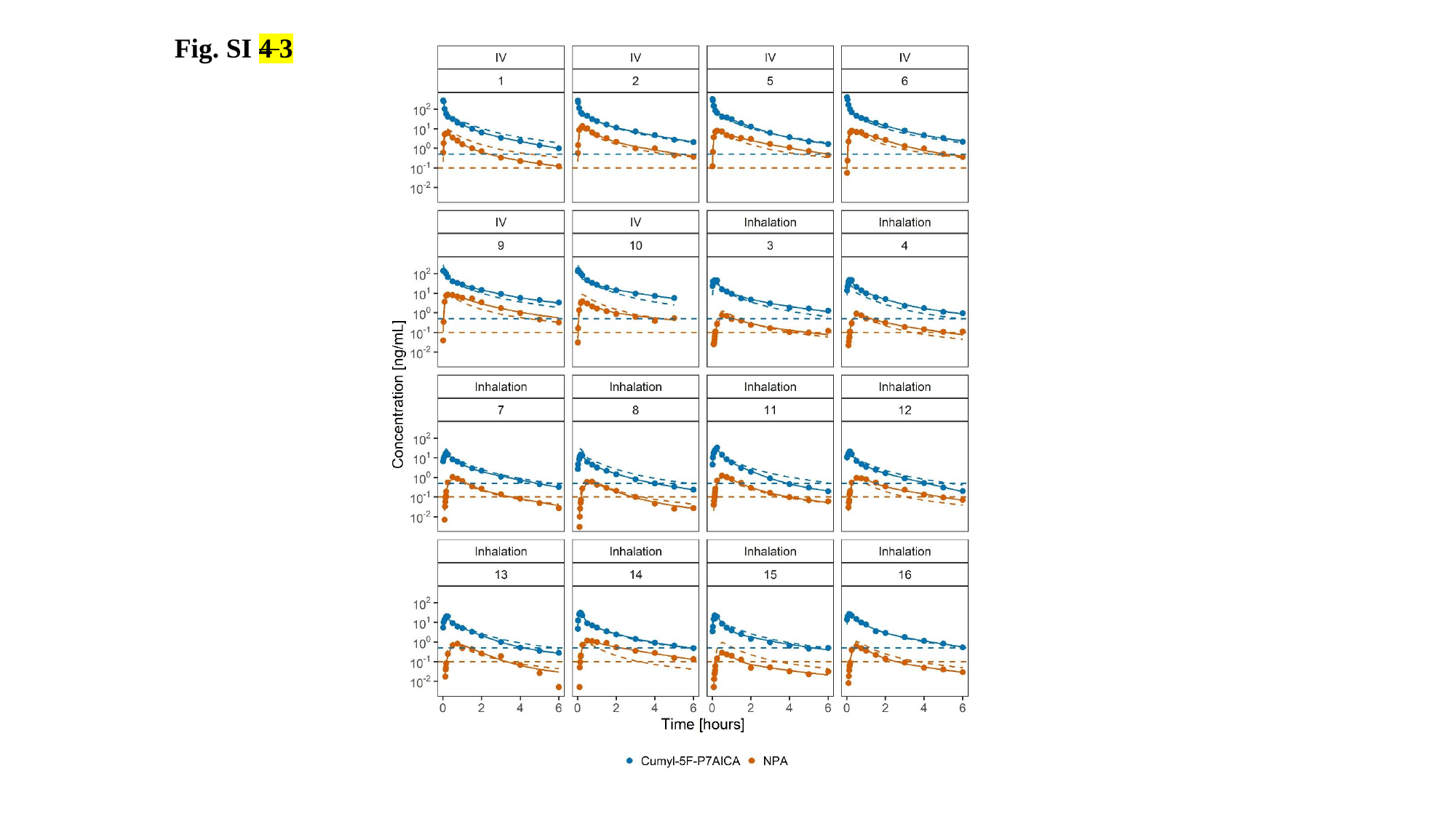

Fig. SI 4 3

Supplement: Supplementary file 4 — Supplementary file4 (PPTX 514 KB) [file 204_2024_3906_MOESM4_ESM.pptx]
